# Assessment of potential drug-drug interactions in patients with hereditary angioedema from the ITACA cohort: simulations from a real-life dataset considering danazol versus berotralstat

**DOI:** 10.3389/fphar.2025.1550133

**Published:** 2025-04-25

**Authors:** Andrea Zanichelli, Dario Cattaneo, Antonio Gidaro, Riccardo Senter, Francesco Arcoleo, Pietro Accardo, Donatella Bignardi, Paolo Borrelli, Caterina Colangelo, Tiziana De Pasquale, Davide Firinu, Francesca Perego, Massimo Triggiani, Giuseppe Spadaro, Chiara Cogliati, Emanuele Bizzi, Valentina Popescu Janu, Maria Domenica Guarino, Paolina Quattrocchi, Luisa Brussino, Oliviero Rossi, Paola Triggianese, Stefano Agolini, Francesco Giardino, Vincenzo Montinaro, Mauro Cancian

**Affiliations:** ^1^ Department of Biomedical Sciences for Health, University of Milan, Milan, Italy; ^2^ UO di Medicina, Centro Angioedema, I.R.C.C.S. Policlinico San Donato, Milano, Italy; ^3^ Unit of Clinical Pathology, Luigi Sacco University Hospital, Milan, Italy; ^4^ Department of Biomedical and Clinical Sciences, Luigi Sacco Hospital, University of Milan, Milan, Italy; ^5^ Department of Systems Medicine, University Hospital of Padua, Padua, Italy; ^6^ UOC di Patologia Clinica e Immunologia, Azienda Ospedaliera Ospedali Riuniti Villa Sofia-Cervello, Palermo, Italy; ^7^ Allergology Unit, IRCCS San Martino Polyclinic Hospital, Genoa, Italy; ^8^ SSD Dermatologia e Allergologia, Ospedale Beauregard, Aosta, Italy; ^9^ Allergy and Clinical Immunology Unit, Azienda Sanitaria Locale Di Pescara, Pescara, Italy; ^10^ Department of Allergology, University Hospital “Maggiore della Carità” of Novara, Novara, Italy; ^11^ Department of Medical Sciences and Public Health, University of Cagliari, Cagliari, Italy; ^12^ Department of Internal Medicine, Istituti Clinici Scientifici Maugeri IRCCS, Milan, Italy; ^13^ Division of Allergy and Clinical Immunology, University of Salerno, Salerno, Italy; ^14^ Department of Translational Medical Sciences, University of Naples Federico II, Naples, Italy; ^15^ Internal Medicine Department, Fatebenefratelli and Sacco Hospitals, Milan, Italy; ^16^ Allergy Unit, Hospital of Civitanova Marche, Civitanova Marche, Italy; ^17^ Department of Clinical and Experimental Medicine, School and Operative Unit of Allergy and Clinical Immunology, University of Messina, Messina, Italy; ^18^ Allergy and Clinical Immunology Unit, Department of Medical Sciences, University of Torino and Mauriziano Hospital, Torino, Italy; ^19^ Immunoallergology Unit, University Hospital of Careggi, Florence, Italy; ^20^ Rheumatology, Allergology and Clinical Immunology, Department of Systems Medicine, University of Roma Tor Vergata, Rome, Italy; ^21^ Department of Clinical Immunology, Azienda Ospedaliera Universitaria Ospedali Riuniti di Ancona, Ancona, Italy; ^22^ Internal Medicine Unit, Azienda Ospedaliero-Universitaria Policlinico “G.Rodolico-San Marco”, Catania, Italy; ^23^ U.O.C. di Nefrologia e Dialisi, Ospedale Generale Regionale “F. Miulli”, Acquaviva delle Fonti (BA), Italy

**Keywords:** hereditary angioedema, C1-inhibitor deficiency, drug-drug interactions, long-term prophylaxis, bradykinin, danazol, berotralstat, ITACA

## Abstract

**Background:**

Danazol is regularly used as a prophylactic treatment in patients with Hereditary angioedema due to C1-inhibitor deficiency (HAE-C1INH). However, this drug is characterized by a risk of drug-drug interactions (DDIs). Berotralstat, the first oral kallikrein inhibitor, has been recently approved for the prevention of HAE attacks. Here, we sought to compare the risk of potential DDIs in real-life HAE patients hypothetically given Danazol or Berotralstat.

**Methods:**

Our clinic’s database was retrospectively reviewed to identify patients diagnosed with HAE who were treated with at least one concomitant medication. The DDIs were assessed using three freely available drug interaction checkers and scored based on their severity. The agreement between the three drug checkers was evaluated using weighted Cohen’s kappa coefficient.

**Results:**

75 HAE patients (64% female, mean age 56 ± 21 years) were considered. They were mainly treated with antihypertensives (37%), hypoglycemic (19%), and hypolipemic agents (17%). Significant discrepancies among the three-drug interaction checkers were found. The first checker identified 18 potential DDIs, all involving Danazol and a statin (simvastatin). The second checker identified, respectively, 66 and 14 DDIs for Danazol (20% severe, regarding Simvastatin and Rivaroxaban) and Berotralstat (0% severe). The third checker identified 49 and 43 DDIs for Danazol (22% severe, regarding Simvastatin) and Berotralstat (0%).

**Conclusion:**

Berotralstat was consistently associated with a reduced risk of DDIs compared with Danazol. A rational assessment of DDIs would help select the best prophylactic treatment for HAE.

## Introduction

Hereditary angioedema (HAE) due to C1-inhibitor (C1INH) deficiency (HAE-C1INH) is an autosomal dominant disease caused by a deficient or dysfunctional C1-inhibitor. This naturally occurring molecule inhibits kallikrein, the protease which liberates bradykinin (BK) from plasma kininogen (Miyata and Horiuchi, 2023). The disease is characterized by painful, recurrent, unpredictable, and debilitating episodes of submucosal and/or subcutaneous tissue swelling, which may be life-threatening if the larynx is involved. HAE treatment options include on-demand therapy for acute attacks, short-term prophylaxis, and long-term prophylaxis (LTP), which should be considered on every visit to achieve complete control of the disease (Maurer et al., 2022).

Danazol and Stanazolol are synthetic attenuated androgens (AA) that effectively prevent HAE attacks. These medications have been used for decades before specific treatments became available (Maurer et al., 2022) and are still used in some countries (Guryanova et al., 2021). However, their side-effect profile can be problematic because it can predispose to cardiovascular and metabolic complaints (Zanichelli et al., 2024; Johnston et al., 2021). For this reason, the latest international WAO/EAACI guidelines suggest their use only as a second choice and propose the switch to a new LTP (Maurer et al., 2022).

The plasma kallikrein inhibitor Berotralstat, which prevents tissue edema elicited by BK during acute episodes of HAE-C1INH, has been approved for prophylaxis to prevent attacks of HAE-C1INH in adults and adolescent patients aged 12 years or older. This drug, easily administered orally like AA, might theoretically represent the most practical alternative to androgens for LTP. Phase III trials and real-world data showed that Berotralstat reduced the number of acute attacks and improved the quality of life in HAE patients (Zuraw et al., 2021; Kiani-Alikhan et al., 2024).

Thanks to recent advances in HAE-C1INH diagnosis and therapy, the life expectancy of those patients is similar to that of the general population (Perego et al., 2020). For this reason, the incidence of comorbidities and the risk of drug-drug interactions (DDIs) due to polypharmacy, which increase with age, can become significant issues even in patients with HAE-C1INH. (Perrella et al., 2024; Goetschi et al., 2024; Chuang et al., 2023; McDonald et al., 2024; Zidan and Awaisu, 2024; Randles et al., 2022).

In this context, both Danazol and Berotralstat may predispose to DDIs. The risk of drug-related adverse events and DDIs with co-medications - including statins, antiepileptics, immunosuppressive agents, and anticoagulants - is well known for Danazol (Stankovic et al., 2010; Andreou and Ledger, 2003; Small et al., 1982; Goulbourne and Macleod, 1981; Krämer et al., 1986; Ross et al., 1986; Zielinski et al., 1987; Watson et al., 1993; Shapiro et al., 1993; Blatt et al., 1996). Even if Berotralstat seems to have a lower propensity to cause DDIs, a recent paper suggested potential interactions with immunosuppressive drugs (Tacrolimus and Prednisone) (Adatia and Magerl, 2024; Gidaro et al., 2024). However, no studies have formally investigated the risk of potential DDIs in HAE-C1INH patients on Berotralstat so far.

To address this issue, we analyzed the potential DDIs between Danazol and associated therapies in a cohort of patients from the Italian network for Hereditary and Acquired angioedema (ITACA). After that, we simulated the potential interactions of Berotralstat in the same patients.

## Materials and methods

### Patient selection and study design

This retrospective cohort study involved adult patients diagnosed with HAE-C1INH referred to ITACA’s angioedema centers from 1979 to 2019, 1 year before Berotralstat became available.

All data used in the study were anonymized following the requirements of the Italian Data Protection Code (leg. decree 196/2003) and the general authorizations issued by the Data Protection Authority.

Enrollment in the ITACA Registry was approved by the ethics committee of the coordinating center (Comitato etico Milano area 1, Italy) on 5 May 2017. According to the Ethics Committee, all patients signed written informed consent.

In the analyses, we considered only co-medications given chronically, excluding drugs given on demand. We also collected the patients’ main demographic characteristics. Subsequently, we assessed the potential DDIs, simulating a hypothetical scenario in which all enrolled patients were treated with Danazol (the most frequent drug used for the prophylaxis of HAE-C1INH before the developing of new therapy) or Berotralstat (a novel prophylactic treatment for HAE).

The risk of DDIs between Danazol or Berotralstat and the co-medications was assessed using INTERcheck WEB (https://intercheckweb.marionegri.it, last access 24 September 2024), Medscape Drug Interaction checker (https://reference.medscape.com/drug-interactionchecker, last access 24 September 2024) and UpToDate (https://www.uptodate.com/drug-interactions, last access 24 September 2024). These checkers were selected because they are freely available.

Based on their severity and clinical relevance, the potential DDIs were classified as red flag (drug combinations that should be avoided), orange flag (drug combinations that may require close monitoring and/or drug dose adjustments due to potentially severe clinical consequences), or yellow flag (drug combinations with minor clinical relevance).

### Statistical analyses

The Kolmogorov-Smirnov test was done to evaluate the data distribution’s normality. Continuous variables were expressed as mean, standard deviation, median, and range. Categorical variables were expressed as absolute numbers or percentages.

The agreement between the three drug checkers was evaluated using weighted Cohen’s kappa coefficient.

## Results

### Patient characteristics

A total of 446 patients from the ITACA dataset with a HAE-C1INH diagnosis were considered. Among the 221 patients who had used AA long-term prophylaxis from 1979 to 2019, 75 had concomitant medications, fulfilled inclusion criteria, and were included in the present study. The main demographic and clinical features are shown in Table 1. The majority were female (64%), with a mean age of 56 ± 21 years, and 36% were over 65. The mean time from onset of symptoms to HAE-C1INH diagnosis was 28 ± 11 years.

**TABLE 1:** Demographic and clinical feature of the patients with hereditary angioedema.

Characteristics	Data
Patients, n	75
Female, n (%)	48 (64%)
Age, years	56 ± 21
Time to HAE diagnosis, year	28 ± 11
Patients with concomitant diseases, %	99%
‐Hypertension, %	69%
‐Dyslipidemia, %	37%
‐Diabetes, %	29%
‐Previous CVD events, %	19%
‐Hepatitis C, %	16%

CVD: cardiovascular; HAE: hereditary angioedema.

60/75 HAE-C1INH patients had been treated only with Danazol as long-term prophylaxis, and this number reflects the fact that, for most of the period under consideration, specific prophylactic medications such as i.v. or s.c. Plasma-derived C1INH, Lanadelumab, and Berotralstat were not yet available. Stanozolol or Tranexamic acid had been previously prescribed to 11 and 4 patients, respectively. Patients receiving tranexamic acid were children under 12 years old who were not taking any other concomitant medications. The most frequent comorbidities were hypertension (diagnosed in 69% of HAE-C1INH patients), dyslipidemia (37%), and diabetes (29%).

### Co-medications

The 75 patients were chronically treated with 161 co-medications in addition to LTP for HAE-C1INH, with a mean of 2,1±1,4 drugs per patient. The most frequent drug classes were anti-hypertensives (37%, mainly amlodipine and doxazosin), hypoglycemic agents (19%, primarily metformin), hypolipemic agents (17%, primarily simvastatin and atorvastatin), diuretics (15%, mainly hydrochlorothiazide) and antithrombotics (6%, primarily clopidogrel).

### Assessment of the potential drug-drug interactions

The weighted kappa value of 0.367 (95% CI: 0.254–0.481) indicates fair agreement (p < 0.001) between INTERCheck web and Medscape, and the same level of agreement is observed at 0.256 (95% CI: 0.16–0.352) between INTERCheck web and UpToDate (p < 0.001). Lastly, a moderate agreement of 0.511 (95% CI: 0.389–0.632) was found between Medscape and UpToDate (p < 0.001).

#### INTERCheck WEB

As shown in Table 2, 18 potential DDIs were identified for Danazol, all categorized as orange-flag DDIs. Among statins, Simvastatin or Atorvastatin could be involved in DDIs, while Rosuvastatin and Fluvastatin did not.

**TABLE 2:** Assessment of potential drug-drug interactions (DDIs) using INTERCHECK WEB.

Danazol	N	Potential adverse event
Total pDDIs	18	
Red-flag pDDIs	0	
Orange-flag pDDIs	18	
Statins	18	Increased effect of atorvastatin or simvastatin by CYP3A4 and/or P-gp inhibition
Yellow-flag pDDIs	0	

Berotralstat	N	Potential adverse event
Total pDDIs	0	
Red-flag pDDIs	0	
Orange-flag pDDIs	0	
Yellow-flag pDDIs	0	

CYP3A4: cytochrome P450 3A4; P-gp: plycoprotein.

No DDIs were identified for Berotralstat.

#### Medscape drug interaction checker

In total, 66 potential DDIs involving Danazol were identified (Table 3) and categorized, respectively, as red-flag (19.7%), orange-flag (25.8%), and yellow-flag DDIs (54.5%). The red-flag DDIs involved Simvastatin (n = 11) or Rivaroxaban (n = 2) due to Danazol’s inhibitory effect on their metabolism, potentially resulting in an increased risk of drug-related toxicity.

**TABLE 3:** Assessment of potential DDIs using MEDSCAPE drug interaction checker.

Danazol	N	Potential adverse event
Total pDDIs	66	
Red-flag pDDIs	13	
‐Simvastatin	11	Increased toxicity of simvastatin by CYP3A4 inhibition
‐Rivaroxaban	2	Increased toxicity of rivaroxaban by CYP3A4 inhibition
Orange-flag pDDIs	17	
‐CCBs	10	Increased effect of amlodipine, verapamil or diltiazem by CYP3A4 inhibition
‐Atorvastatin	7	Increased effect of atorvastatin by CYP3A4 inhibition
Yellow-flag pDDIs	36	
‐Metformin	18	Increased effects of metformin by PD synergism
‐ASA	4	Decreased effect of danazol by CYP3A4 inhibition
‐Losartan	4	Increased effect of losartan by CYP3A4 inhibition
‐Insulin	3	Increased effects of insulin by PD synergism
‐Repaglinide	2	Increased effect of repaglinide by CYP3A4 inhibition and PD synergism
‐Fenofibrate	2	Increased effect of fenofibrate by CYP3A4 inhibition
‐Glimepiride	1	Increased effects of glimepiride by PD synergism
‐DPP IV inhibitors	2	Increased effects of vildagliptin or sitagliptin by PD synergism

Berotralstat	N	Potential adverse event
Total pDDIs	14	
Red-flag pDDIs	0	
Orange-flag pDDIs	14	
‐Carvedilol	1	Increased effect of both drugs by mutual inhibition of P-gp
‐CCBs	2	Increased effect of diltiazem or verapamil by P-gp inhibition
‐Atorvastatin	7	Increased effect of both drugs by mutual inhibition of P-gp
‐DOACs	2	Increased effect of dabigatran or apixaban by P-gp inhibition
‐DPP IV inhibitors	2	Increased effect of sitagliptin or linagliptin by P-gp inhibition
Yellow-flag pDDIs	0	

CYP3A4: cytochrome P450 3A4; P-gp: plycoprotein; PD: pharmacodynamic; ASA: acetilsalycilc acid including lysine salts; DOACS: direct oral anticoagulants; DPP: dipeptidyl dipeptidases; CCB: calcium channel blockers.

Only 14 potential DDIs were identified for Berotralstat, and all scored as orange-flag DDIs. The most frequent DDI concerned Atorvastatin, which acted both as victim and perpetrator because of the mutual inhibitory effect of both drugs on p-glycoprotein.

#### UpToDate drug interaction checker

As shown in Table 4, 49 potential DDIs were identified for Danazol and categorized as red-flag (22,4%) or orange-flag DDIs (77,6%). All the red-flag DDIs involved Simvastatin as a victim of the inhibitory effect of Danazol, potentially resulting in an increased risk of drug-related toxicity. The large majority of orange-flag DDIs (81,7%) involved hypoglycemic agents, whose therapeutic effect could be diminished by concomitant Danazol administration.

**TABLE 4:** Assessment of potential DDIs using UpToDate drug interactions checker.

Danazol	N	Potential adverse event
Total pDDIs	49	
Red-flag pDDIs	11	
‐Simvastatin	11	Increases toxicity of simvastatin by CYP3A4 inhibition
Orange-flag pDDIs	38	
‐Metformin	18	reduced effect of metformin by PD antagonism
‐Atorvastatin	7	Increased effect of atorvastatin by CYP3A4 inhibition
‐Insulin	3	Increased effects of insulin by PD synergism
‐Sulphanyluree	3	Reduced effect of glibenclamide or glimepride by PD antagonism
‐Repaglinide	2	Reduced effect of repaglinide by PD antagonism
‐SGLT2 inhibitors	2	Reduced effect of dapaglifozin or empaglifozin by PD antagonism
‐DPP IV inhibitors	3	Reduced effect of sitagliptin, vidagliptin, linagliptin by PD antagonism
Yellow-flag pDDIs	0	

Berotralstat	N	Potential adverse event
Total pDDIs	43	
Red-flag pDDIs	0	
Orange-flag pDDIs	33	
‐Statins	18	Increased effect of simvastatin or atorvastatin by CYP3A4 inhibition
‐CCBs	12	Increased effect of amlodipine, diltiazem, verapamil, or lercanidipine by CYP3A4 inhibition
‐DOACs	3	Increased effect of rivaroxaban or apixaban by CYP3A4 inhibition
Yellow-flag pDDIs	10	
‐Clopidogrel	6	Increased exposure of berotralstat by BCRP Inhibition
‐Repaglinide	2	Increased exposure of repaglinide by CYP3A4 inhibition
‐Carvedilol	1	Increased exposure of carvedilol by P-gp inhibition
‐Propafenone	1	Increased exposure of both drugs by mutual inhibition of P-gp/CYP3A4

CYP3A4: cytochrome P450 3A4; P-gp: plycoprotein; PD: pharmacodynamic; DOACs: direct oral anticoagulants; DPP: dipeptidyl dipeptidase; CCB: calcium channel blocker; SGLT: sodium-glucose transport protein; BCRP: breast resistance cancer protein.

Overall, 43 potential DDIs were identified for Berotralstat, categorized as orange-flag (76,7%) or yellow-flag DDIs (23,3%). Most of the orange-flag DDIs were related to the inhibitory effect of Berotralstat on CYP3A4, potentially increasing the exposure and/or effects of statins, calcium channel blockers, or direct oral anticoagulants.

## Discussion

Using a real-world dataset of HAE-C1INH patients from the ITACA network, we documented in this simulation that potential DDIs with additional chronic treatments in patients on LTP are lower with Berotralstat than with Danazol. Remarkably, no red-flag DDIs have been identified for Berotralstat, whereas Danazol was associated with nearly 20% of red-flag DDIs by two out of the three drug interaction checkers used.

This is not an unexpected finding, as Danazol is a moderate/strong inhibitor of intestinal and hepatic CYP450 3A4, 3A5 (CYP3A4/5), and 2D6 (CYP2D6), the isoenzymes responsible for the metabolic clearance of the large majority of drugs available on the market (Adatia and Magerl, 2024; Lee et al., 2012). This accounts for why clinically relevant DDIs have been extensively reported involving Danazol as the perpetrator and several drugs as victims, such as statins, antiepileptics, immunosuppressive drugs, anticoagulants, etc. (Stankovic et al., 2010; Andreou and Ledger, 2003; Small et al., 1982; Goulbourne and Macleod, 1981; Krämer et al., 1986; Ross et al., 1986; Zielinski et al., 1987; Watson et al., 1993; Shapiro et al., 1993; Blatt et al., 1996). Indeed, the Danazol-related inhibition of drug metabolism increases the exposure and the activity of most of the co-medications and the risk of drug-related toxicity (or the risk of reduced drug efficacy if the co-medication is a prodrug that requires CYP3A4 to be converted in the active metabolite). Conversely, according to the available literature, Berotralstat has a lower propensity to be involved in DDIs, being only a moderate inhibitor of CYP3A4 and CYP2D6 (Adatia and Magerl, 2024; EMA, 2024). The drug is also a substrate and a weak inhibitor of P-glycoprotein and BCRP, two drug transporters regulating drug distribution in the body compartments (Adatia and Magerl, 2024). As a result, the three-drug interaction checkers consistently reported not only fewer DDIs between Berotralstat and the co-medications but, most importantly, a reduced number of DDIs scored as potentially clinically relevant (red-flag or orange-flag) compared with Danazol. In particular, the red-flag DDIs of Danazol involved the potential risk of hepatic and muscular toxicity or the risk of bleeding if co-administered, respectively, with Simvastatin or with Rivaroxaban; for both drugs, the orange-flag DDIs involved a potential risk to increase the exposure and the effects of anti-hypertensives (calcium channel blockers, beta-blockers) and other statins (Atorvastatin).

As an additional finding of the present study, we observed significant heterogeneity and inconsistencies in the number and the severity of potential DDIs involving Danazol or Berotralstat reported by the three-drug interaction checkers used in our simulation. The hypoglycemic agents give a critical example: according to INTERCheck, no DDIs are expected between Danazol and these drugs; MEDSCAPE reports that Danazol may increase the effect of Metformin, dipeptidyl peptidase IV inhibitors, or glinides, whereas for UpToDate, Danazol is a hyperglycemia-associated agent who could diminish the therapeutic effects of antidiabetic agents, glifozins included. Such inconsistencies between the drug interaction checkers, which have been extensively described in the literature (Iversen et al., 2022; Carollo et al., 2024a; Carollo et al., 2024b; Günay et al., 2022; Roca and Roca, 2022; Monteith and Glenn, 2019), may be related to the lack of standardized methods and criteria used to classify DDIs or, in the case of new drugs like Berotralstat, by the lack of data on their use in real-life settings. Taken together, these results highlight the challenges that healthcare professionals need to face in their daily clinical practice when assessing the risk of DDIs and the safety of medications. The availability of multidisciplinary teams involving clinical pharmacologists/clinical pharmacists might help to address this issue by removing inappropriate drugs and/or guiding in the interpretation of the clinical relevance of potential DDIs when data from interaction checkers are conflicting, as we previously reported in people living with HIV, and in patients with mycobacterial or fungal infections (Cattaneo et al., 2023a; Cattaneo et al., 2020; Cattaneo et al., 2024; Cattaneo et al., 2023b).

Some important information can be retrieved from our study despite the limited overlap between the three-drug interaction checkers. For instance, all checkers consistently reported DDIs between danazol simvastatin (considered a red flag DDIs by 2 out of the three checkers) and, to a lesser extent, with atorvastatin. These DDIs, which are likely to become even more clinically relevant when these statins are used at high doses (i.e., simvastatin at 80 mg and atorvastatin at 40 mg), may require a close monitoring of transaminases and creatinine phosphokinases. Other important DDIs involving danazol may be related to the opposite and poorly predictable effect of this drug on hypoglycemic agents and insulin. Indeed, danazol may reduce the effect of metformin, sulphonylurea, SGLT2, and DPP IV inhibitors by pharmacodynamic antagonism, increasing at the same time the effect of insulin by PD synergism. The take-home message is that diabetic patients undergoing LTP with danazol require strict, intensive metabolic control. The potential DDIs between danazol or berotralstat and CCBs, although scored as orange-flag DDIs, might be less clinically relevant considering that our patients are used to monitoring BP regularly. Conversely, the orange-flag DDIs involving berotralstat and DOACs may be more challenging, possibly requiring proper drug dose adjustments to avoid the risk of bleeding.

This simulation used data collected mainly from patients diagnosed with HAE-C1INH referred to the Sacco Hospital in Milan. Therefore, selection bias and/or underestimating potential DDIs cannot be ruled out. For instance, nearly 20% of HAE-C1INH patients from our cohort were treated with proton pump inhibitors (PPIs), possibly reflecting the local approach to prevent potential gastric side effects, with no detailed information in the database on the timing of administration of these drugs (i.e., chronic versus on demand) or the type of PPI. This prevents a proper assessment of the pDDIs because, even if INTERCheck WEB and UpToDate Drug Interaction Checker do not report any DDIs between PPIs and Danazol or Berotralstat, for MEDSCAPE drug interaction checker Omeprazole, Rabeprazole, or Esomeprazole co-administration might result in yellow-flag DDIs with Danazol (increased effect of the PPIs by CYP3A4 inhibition). In contrast, Pantoprazole co-administration may result in an orange-flag DDI with Berotralstat (increased effect of Berotralstat by BCRP inhibition).

In conclusion, despite the significant discrepancies among the three-drug interaction checkers, Berotralstat was consistently associated with a reduced risk of potential DDIs compared with Danazol, which has been extensively used in the past for LTP in Italy and is still extensively used in countries with limited access to innovative, pathway-specific prophylactic treatments. A rational assessment of DDIs would contribute to better selecting the best prophylactic treatment for HAE-C1INH patients.

## Data Availability

The raw data supporting the conclusions of this article will be made available by the authors, without undue reservation.
